# Effect of diclofenac suppository on pain control during flexible cystoscopy-A randomized controlled trial

**DOI:** 10.12688/f1000research.9519.1

**Published:** 2016-12-08

**Authors:** Mehwash Nadeem, M Hammad Ather

**Affiliations:** 1 Aga Khan University, Karachi, Pakistan

**Keywords:** diclofenac suppository, pain control, flexicystoscopy, office urology

## Abstract

**TRIAL DESIGN: **To compare the difference in pain score during flexible cystoscopy between patients undergoing the procedure with plain lubricating gel  only and plain gel with diclofenac suppository in a randomized control trial.

**METHODS**:  A total of 60 male patients with an indication of flexible cystoscopy were enrolled in a prospective, randomized controlled study. Patients were randomized in two groups. In group “A”, patients received diclofenac suppository one hour prior to the procedure while group “B” did not receive diclofenac suppository. Both groups received 10 ml of intra-urethral  plain gel for lubrication during flexible cystoscopy. Pain score was recorded immediately after the procedure using the visual analogue scale (VAS). Pre- and post-procedure pulse rate and systolic blood pressure was also recorded. Statistical analyses were performed using chi-square test and student t-test. Regression analysis was performed to address the confounding variables.

**RESULTS**: Both groups were comparable for variables including age, duration of procedure, level of operating surgeon and indication of procedure. Most common indication for flexible cystoscopy was removal of double J stent. There was a statistically significant difference in the mean pain score between two groups ( *p* = 0.012).  The difference in post-procedure mean pulse rate in the two groups was statistically significant ( *p*= 0.01) however there was no difference observed in mean post procedure systolic blood pressure. Regression analysis showed that none of the confounding variables were significantly affecting pain perception.

**CONCLUSIONS:** Intra rectal diclofenac suppository is simple and effective pre-emptive analgesia. We recommend its routine use during flexible cystoscopy for better pain control.

## Introduction

The earliest reported use of flexible endoscope for examination of bladder neck was by Tsuchida and Sugawara ^1^. It is now one of the most commonly performed diagnostic as well as therapeutic urologic interventions ^2^. Pain associated with cystoscopy varies from patient to patient and there is continuous effort using various methods to reduce pain during and after the procedure to improve patient compliance for flexible cystoscopy. The majority of patients require local anesthesia or lubricant solution only but some patients may require intravenous sedation ^3^ or inhalation analgesia (nitrous oxide) ^4^. Factors contributing to severity of pain include: lubrication, use of topical anesthesia and duration of cystoscopy ^5– 7^ but the available evidence for best practice in terms of treatment is continuously evolving ^8^. The important issues regarding the correct use of intra-urethral gels are, for the most part, left to individual preference ^9^. Effect of different intra-urethral gels, their dosage, temperature and time of instillation on pain perception has been evaluated in literature. In a randomized control trail, 2% lidocaine gel in two different doses (10 and 20 ml) and plain lubricating gels were found to be equally effective for pain control during flexible cystoscopy *(p=*0.406) ^10^. Pain perception with use of lidocaine versus plain lubricating gel is less as reported in a meta-analysis by Aaronson *et al.* ^11^ while another meta-analysis by Patel *et al.* has reported no statistical difference among the two gels for pain control ^12^. In a study by Komiya *et al.*, oral zaltoprofen has been used as pre-emptive analgesia for rigid cystoscopy and it has been proved to provide better pain control than 2% lidocaine gel alone (11.35 versus 13.69 with a difference of pain score -2.8, p-value 0.0087) ^13^. Intra-rectal diclofenac suppository administration used by Irer *et al.* has a proven role to reduce pain and improve patients’ tolerance of trans rectal ultrasound-guided prostate biopsy ^14^.

Diclofenac is an anti-inflammatory drug with local and systemic effects; the local effects include reducing the impact of pain mediators. The diclofenac suppository in comparison to the oral has a rapid onset and a slower rate of absorption. The maximal plasma level Is reached within 2 hours, and is maintained for up to 12 hours and that forms the basis of using suppository rather than oral NSAID in our study ^15^. In the current study we have attempted to assess the use of diclofenac suppository as a pre-emptive analgesia during flexible ureteroscopy.

## Methodology

### Study protocol, patient recruitment and randomization

The Ethical Review Committee of the Aga Khan University and the Clinical Trial Unit approved the study protocol. The study was registered at www.clinicaltrials.gov (ClinicalTrials.gov identifier: NCT01812928). This trial was conducted at the surgical day care unit from February 2013 to July 2013.

**Figure 1.**
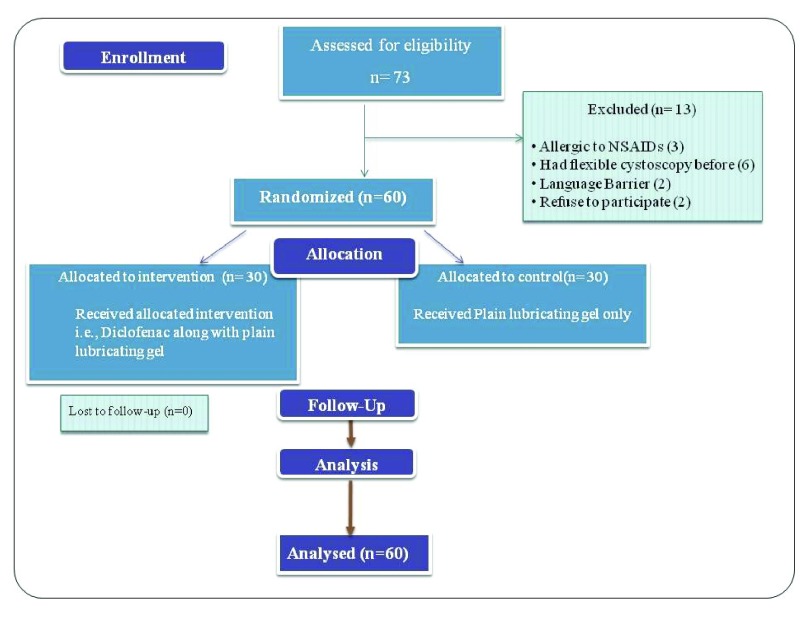
CONSORT statement describing the details eligibility, allocation, follow up and analysis of the patients.

Details of recruitment and flow of study has been demonstrated as CONSORT flow diagram. ( Figure 1). The principal investigator of this study obtained the written consent from all the qualified patients before randomization. All male patients of 18 years of age and older with indication for flexible cystoscopy, were assessed for recruitment in the trial. We included all adult males who attended for evaluation of hematuria or lower urinary tract **s**ymptoms and those for removal of double J ureteral stent. All patients undergoing the procedure had a urinalysis and culture to exclude UTI. Patients were requested to empty the bladder immediately prior to the procedure or within 30 minutes. Prior to the procedure, patients were explained the visual analog scale (VAS; score zero means no pain and 10 means worst pain). Eligible patients were randomized by a computer-generated list and sealed envelopes. Patients were randomized into either Group A (those patients who received diclofenac suppository prior to procedure) or Group B (those patients who did not receive diclofenac suppository prior to procedure) using a web-based random number generator (RANDOM.ORG, Dublin, Ireland; https://www.random.org). Diclofenac suppository (100 mg) was administered rectally 1 hour prior to the procedure in the pre-operative area. Both groups received 10 ml of plain lubricating gel immediately before the procedure for the purpose of lubrication.

**Figure 2.**
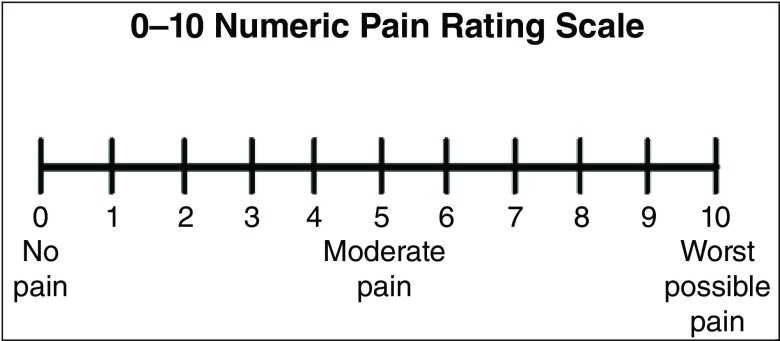
Numeric pain rating on a scale of 1–10.

The procedure was performed at the surgical day care unit in supine position by a consultant urologist or senior urology resident (residency year 5 and 6) that was blinded to the randomization group. A second resident immediately following the procedure, collected data (pain score) in the operating room. The VAS consists of a straight line with the endpoints defining extreme limits such as ‘no pain at all’ and ‘pain as bad as it could be’ ^16^. The investigator was blinded to the group (independent assessor). Operative time was recorded from the operating room time log. Pre- and post-procedure pulse rate and blood pressure were recorded for all participants.

### Data analysis

Data was analyzed using SPSS™ version 17.0. Results were described in terms of mean and standard deviation for age, duration of procedure and pain score while frequency and percentage were mentioned for categorical variables. The student t-test (independent samples, one-tailed) was used to determine statistical significance of VAS for pain between group A and B. Confounder and effect modifiers i.e. age, level of the person performing procedure, indication for procedure and duration of procedure were analyzed using linear regression analysis. *p*-value of <0.05 was considered as statistically significant.

## Results

Raw data for ‘Effect of diclofenac suppository on pain control during flexible cystoscopy-A randomized controlled trial’, 2016 Group A: Diclofenac + Gel; Group B: Gel alone; Indication: 1= LUTS, 2= Haematuria, 3= JJ Stent removal. Click here for additional data file. Copyright: © 2016 Nadeem M and Ather MH 2016 Data associated with the article are available under the terms of the Creative Commons Zero "No rights reserved" data waiver (CC0 1.0 Public domain dedication).

Seventy-three patients were evaluated for inclusion in the study. A total of sixty patients were recruited in the trial and analyzed. The mean age was 46.75 ± 16.12 years (IQR: 18–80). The most common indication for flexible cystoscopy was removal of double J Stent (n= 38, 63.3%), others were for evaluation of hematuria (16, 26.7%) and lower urinary tract symptoms (6, 10%). Year 5 and 6 urology residents performed the majority of the procedures (n= 56). Mean duration of the procedure was 5.52 ± 2.13 minutes (IQR: 2–10 minutes). On the 11 point VAS the mean pain score was 3.63 with a standard deviation of 1.46 for the entire group (IQR: 0 – 7). The highest pain score was of 7 on VAS reported by only one patient from group B.

The mean age of the patients in groups A and group B were 48.53 ± 17.81 years and 44.97 ± 14.31 years respectively and there was no statistically significant difference ( *p*= 0.53). The pre-procedure pulse and systolic blood pressures were comparable in both groups. Mean duration of procedure in group A was 5.76 ± 2.25 minutes and in group B was 5.28 ± 2.00 minutes. This difference in duration was not statistically significant ( *p*=0.82). Indications for the procedure and level of operating surgeon were also comparable between the groups.

Mean pain score in group A was 3.16 ± 1.53 and in group B was 4.10 ± 1.24. This difference in the mean pain score was found to be statistically significant ( *p*= 0.012). None of our patients required additional analgesia in either group. The difference in post-procedure pulse rate was found to be statistically significant ( *p*=0.01) between groups however no statistically significant difference ( *p*=0.15) was observed in systolic blood pressure between two groups ( Table 1).

**Table 1.** Basic demographic profile of the patients in the two groups.

Parameters	Group A	Group B	p value
**Age (years)** Mean ± SD	48.53 ± 17.8	44.97 ± 14.3	0.53
**Duration (min)** Mean ± SD	5.76 ± 2,25	5.28 ± 2.0	0.82
**Indications**			
JJ stent removal	17	21	
Evaluation of hematuria	10	6	0.497
Evaluation of LUTS	3	3	
**Level of operating surgeon**			
Consultant urologist	2	2	
Senior urology resident	28	28	0.694
**Post-procedural pulse/min** Mean ± SD	73.5 ± 4.1	76.4 ± 3.8	**0.01**
**Post-procedural systolic pressure** Mean ± SD	129.3	130.1	0.15
**Pain score on VAS** Mean ± SD	3.16 ± 1.53	4.10 ± 1,24	**0.012**

Linear regression analysis was performed. None of the confounding factors (including age, indication for procedure, level of operating surgeon and duration of procedure) was found to have significant impact on the outcome parameter (r ^2^ = 0.026, standard error of estimate= 1.479; Table 2).

**Table 2.** Multiple linear regression analysis of factors associated with pain. **Model summary (a)**

Model	R	R square	Adjusted R square	Std error of the estimate
1	0.163 ^*^	0.026	-0.026	1.47961

^*^ Predictor: (constant), indication for procedure

**Table:** **Duration of procedure (b)**

	Coefficients*				
Model	Unstandardized coefficient		Standardized coefficient	t	Sig	95% CI for B	
	B	Std. Error	Beta			Lower bound	Upper Bound
1 (constant)	4.548	1.055		4.301		2.434	6.662
Group	-0.251	0.387	-0.086	-0.647	0.520	-1.027	0.526
Duration of procedure	-0.101	0.096	-0.147	-1.048	0.299	-0.293	0.092
Indication for procedure	0.007	0.233	0.004	0.032	0.975	-0.460	0.475

## Discussion

We examined the effect of pre-emptive analgesia on pain perception during flexible cystoscopy and found out that diclofenac suppository significantly reduces pain when administered as pre-emptive analgesia before flexible cystoscopy.

Randomized studies by Patel *et al.* ^12^ regarding use of lidocaine versus plain gel, which included 817 patients, showed that intra urethral lidocaine gel had no statistical effect on pain on a 100-point VAS scale (95% CI, -9.6 to 0.385). This meta-analysis challenged the commonly held belief among clinicians that intra urethral lidocaine gel is more efficacious than plain gel for decreasing pain during flexible cystoscopy ^12^. In contrast to the findings of Patel *et al.* ^12^, Cornel *et al.* observed slightly less pain (statistically non significant) in the test group and pain perception was the same between patients with past experience of cystoscopy and initial cystoscopy ^17^. To avoid this bias, we kept very strict inclusion criteria and excluded all the patients with previous experience of flexible cystoscopy.

The present study has demonstrated significant reduction in pain perception during flexible cystoscopy in male patients with use of diclofenac suppository as pre-emptive analgesia. Sample size was calculated *a priori* to detect the effect, according to Lwanga *et al.* ^18^ We followed stringent criteria for enrollment of patients in this trial to eliminate confounding factors for pain. Computer generated sequences were used for randomization in order to give equal chance of being selected in either group to all recruited patients.

Flexible cystoscopy is often performed repeatedly in particular during the follow up of urothelial cancer. As repeated cystoscopy did not increase the patient's tolerability to pain associated with cystoscopy, Muezzinoglu noted the need for more effective anesthesia to improve tolerability during the procedure and maintain quality of life of the patients under long-term follow-up with repeated cystoscopies ^19^. Till date various techniques have been used to ameliorate the perception of pain during flexible cystoscopy. Use of NSAID as pre-emptive analgesia has been tested for various surgical procedures ^20, 21^. Komiya and co-workers examined the effect of anti-inflammatory drug (NSAID) zaltoprofen that inhibits the generation of prostaglandins as well as the pain induced by bradykinin during rigid cystoscopy ^13^. The mean age of the patients in their study was 69.3+/- 8.2 (range: 41–83) while in our study we had relatively younger study subjects (mean age+/- SD, range: 46.75+/-16.1 years, 18–80 years) who are presumably more anxious with lower pain threshold. Despite this fact, diclofenac suppository significantly improved the pain perception and proved to be effective regardless of age on regression analysis. Another matter of debate is the statistical method used in the study by Komiya *et al.* ^13^ where they used a “one sample Wilcoxon test” for comparing the two groups which is rather an inappropriate test to demonstrate the effect. The one-Sample Wilcoxon signed-rank test is a non-parametric alternative to a one-sample t-test. The test determines whether the median of the sample is equal to some specified value. Data should be distributed symmetrically about the median. In the present study we have used regression analysis, which is a more stringent method to demonstrate the effect.

In our study, we used diclofenac suppository as pre-emptive analgesia. The pharmacokinetics of the suppository form is quite different from the orally administered agent. It acts as an anti-inflammatory drug both locally and systemically, by minimizing the effects of local mediators involved in the pain response. Diclofenac has been marketed internationally since 1973 and is currently available in oral, rectal, parenteral and topical preparations ^15^. The efficacy of the diclofenac suppository is due to more rapid onset of effect, and a slower rate of absorption (it takes approximately 4.5 hours for complete absorption) than oral enteric-coated tablets. The maximal plasma level is attained within 2 hours, and it is maintained for up to 12 hours ^15^. The terminal half-life of diclofenac in plasma is 1 to 2 hours. The major route of excretion is the urine (~60%) and a small percentage through bile in the feces ^22^. Its role has proven to be effective for pain control during trans rectal ultra sound guided prostate biopsy in study by Haq *et al.* ^23^. In a case control the investigators noted that it is a simple and safe method. While Irer *et al.* ^14^ showed additional benefit of using lidocaine gel for pain control during the same procedure but statistical significance of this study is in question due to its smaller sample size.

In the present study, appropriate sample size, stringent criteria for recruitment, computer generated randomization, proper statistical methods and analysis has increased the scientific rigor. This was not a placebo controlled as various per rectally medications or “dummy drugs” may have some local inflammatory effect.

## Conclusion

Intra rectal diclofenac suppository is a simple and effective method to reduce pain during flexible cystoscopy regardless of age. We recommend its routine use for better tolerability of pain and to increase patient’s compliance.

## Data availability

The data referenced by this article are under copyright with the following copyright statement: Copyright: © 2016 Nadeem M and Ather MH

Data associated with the article are available under the terms of the Creative Commons Zero "No rights reserved" data waiver (CC0 1.0 Public domain dedication). 

*F1000Research*: Dataset 1. Raw data for ‘Effect of diclofenac suppository on pain control during flexible cystoscopy-A randomized controlled trial’, 2016, 10.5256/f1000research.9519.d145268 ^24^

## Supplementary material

CONSORT checklist and original study protocol.

## References

[1] Tsuchida S Sugawara H: A new flexible fibercystoscope for visualization of the bladder neck. *J Urol.* 1973;109(5):830–1. 4699678 10.1016/s0022-5347(17)60554-8

[2] Beaghler M Grasso M 3rd Loisides P: Inability to pass a urethral catheter: the bedside role of the flexible cystoscope. *Urology.* 1994;44(2):268–70. 10.1016/S0090-4295(94)80148-7 8048205

[3] Song YS Song ES Kim KJ: Midazolam anesthesia during rigid and flexible cystoscopy. *Urol Res.* 2007;35(3):139–42. 10.1007/s00240-007-0091-7 17415555

[4] Calleary JG Masood J Van-Mallaerts R: Nitrous oxide inhalation to improve patient acceptance and reduce procedure related pain of flexible cystoscopy for men younger than 55 years. *J Urol.* 2007;178(1):184–8; discussion 188. 10.1016/j.juro.2007.03.036 17499771

[5] Kobayashi T Nishizawa K Ogura K: Is instillation of anesthetic gel necessary in flexible cystoscopic examination? A prospective randomized study. *Urology.* 2003;61(1):65–8. 10.1016/S0090-4295(02)02002-2 12559267

[6] Kobayashi T Nishizawa K Mitsumori K: Instillation of anesthetic gel is no longer necessary in the era of flexible cystoscopy: a crossover study. *J Endourol.* 2004;18(5):483–6. 10.1089/0892779041271535 15253827

[7] Herr HW Schneider M: Immediate versus delayed outpatient flexible cystoscopy: final report of a randomized study. *Can J Urol.* 2001;8(6):1406–8. 11788018

[8] Soomro KQ Nasir AR Ather MH: Impact of patient's self-viewing of flexible cystoscopy on pain using a visual analog scale in a randomized controlled trial. *Urology.* 2011;77(1):21–3. 10.1016/j.urology.2010.08.012 20974485

[9] Tzortzis V Gravas S Melekos MM: Intraurethral lubricants: a critical literature review and recommendations. *J Endourol.* 2009;23(5):821–6. 10.1089/end.2008.0650 19397430

[10] McFarlane N Denstedt J Ganapathy S: Randomized trial of 10 mL and 20 mL of 2% intraurethral lidocaine gel and placebo in men undergoing flexible cystoscopy. *J Endourol.* 2001;15(5):541–4. 10.1089/089277901750299366 11465336

[11] Aaronson DS Walsh TJ Smith JF: Meta-analysis: does lidocaine gel before flexible cystoscopy provide pain relief? *BJU Int.* 2009;104(4):506–9; discussion 9–10. 10.1111/j.1464-410X.2009.08417.x 19239453

[12] Patel AR Jones JS Babineau D: Lidocaine 2% gel versus plain lubricating gel for pain reduction during flexible cystoscopy: a meta-analysis of prospective, randomized, controlled trials. *J Urol.* 2008;179(3):986–90. 10.1016/j.juro.2007.10.065 18206920

[13] Komiya A Endo T Kobayashi M: Oral analgesia by non-steroidal anti-inflammatory drug zaltoprofen to manage cystoscopy-related pain: a prospective study. *Int J Urol.* 2009;16(11):874–80. 10.1111/j.1442-2042.2009.02384.x 19780869

[14] Irer B Gulcu A Aslan G: Diclofenac suppository administration in conjunction with lidocaine gel during transrectal ultrasound-guided prostate biopsy: prospective, randomized, placebo-controlled study. *Urology.* 2005;66(4):799–802. 10.1016/j.urology.2005.04.053 16230141

[15] Idkaidek NM Amidon GL Smith DE: Determination of the population pharmacokinetic parameters of sustained-release and enteric-coated oral formulations, and the suppository formulation of diclofenac sodium by simultaneous data fitting using NONMEM. *Biopharm Drug Dispos.* 1998;19(3):169–74. 10.1002/(SICI)1099-081X(199804)19:3<169::AID-BDD83>3.0.CO;2-C 9570000

[16] Freyd M: The graphic rating scale. *J Educ Psychol.* 1923;14(2):83–102. 10.1037/h0074329

[17] Cornel EB Oosterwijk E Kiemeney LA: The effect on pain experienced by male patients of watching their office-based flexible cystoscopy. *BJU Int.* 2008;102(10):1445–6. 10.1111/j.1464-410X.2008.07777.x 18540935

[18] Lwanga SK Lemeshow S: Sample size determination in health studies. WHO: Geneva,1991 Reference Source

[19] Müezzinoglu T Ceylan Y Temeltaş G: Evaluation of pain caused by urethrocystoscopy in patients with superficial bladder cancer: a perspective of quality of life. *Onkologie.* 2005;28(5):260–4. 10.1159/000085110 15867482

[20] Nagatsuka C Ichinohe T Kaneko Y: Preemptive effects of a combination of preoperative diclofenac, butorphanol, and lidocaine on postoperative pain management following orthognathic surgery. *Anesth Prog.* 2000;47(4):119–24. 11432176 PMC2149035

[21] Buvanendran A Kroin JS: Multimodal analgesia for controlling acute postoperative pain. *Curr Opin Anaesthesiol.* 2009;22(5):588–93. 10.1097/ACO.0b013e328330373a 19606021

[22] Zmeili S Hasan M Najib N: Bioavailability and pharmacokinetic properties of 2 sustained-release formulations of diclofenac sodium, Voltaren vs inflaban: effect of food on inflaban bioavailability. *Int J Clin Pharmacol Ther.* 1996;34(12):564–70. 8996854

[23] Haq A Patel HR Habib MR: Diclofenac suppository analgesia for transrectal ultrasound guided biopsies of the prostate: a double-blind, randomized controlled trial. *J Urol.* 2004;171(4):1489–91. 10.1097/01.ju.0000115706.19605.e4 15017205

[24] Ather MH Nadeem M: Dataset 1 in: Effect of diclofenac suppository on pain control during flexible cystoscopy-A randomized controlled trial. *F1000Research.* 2016 Data Source 10.12688/f1000research.9519.1 PMC5321120 28299180

