# Supplementary material for: Effect of diclofenac suppository on pain control during flexible cystoscopy-A randomized controlled trial
Source: F1000Res. 2016 Dec 8;5:2834. [Version 1] doi: 10.12688/f1000research.9519.1 (PMC5321120; doi:10.12688/f1000research.9519.1)
Supplement: Supplementary file 2 [file f1000research-5-10254-s0001.tgz › cbb9c8fd-4ae6-400f-8a20-f642167cca6f.zip!/CONSORT Checklist for DUF.docx]

**CONSORT Checklist**

- 1a. Title
- 1b. Abstract
- 2a. Background
- 2b. Objective
- 3. Methods
- 3a. Trial design
- 3b. Changes to trial design
- 4a. Participants
- 4b. Study settings
- 5. Interventions
- 6a. Outcomes
- 6b. Changes to outcomes
- 7a. Sample size
- 7b. Interim analyses and stopping guidelines
- 8a. Randomization: Sequence generation
- 8b. Randomization: Type
- 9. Randomization: Allocation concealment method
- 10. Randomization: Implementation
- 11a. Blinding
- 11b. Similarity of Interventions
- 12a. Statistical methods
- 12b. Additional analysis

Results

- 13a. Participant Flow
- 13b. Losses and Exclusions
- 14a. Recruitment
- 14b. Reason for stopped trial
- 15. Baseline data
- 16. Number analyzed
- 17a. Outcomes and estimation
- 17b. Binary outcomes
- 18. Ancillary analyses
- 19. harms

Discussion

- 20. Limitations
- 21. Generalisability
- 22. Interpretation
- 23. Registration
- 24. Protocol
- 25. Funding

1a) Title:

Effect of Diclofenac Suppository on Pain Control during Flexible Cystoscopy-A Randomized Controlled Trial.

1b) Abstract:

**OBJECTIVE:** To compare the difference in pain score during flexible cystoscopy between patients undergoing the procedure with plain gel only and plain gel with diclofenac suppository.

**METHODS**: A total of 60 male patients with an indication of flexible cystoscopy were enrolled in a prospective, randomized controlled Study. Patients were randomized in two groups. In group “A”, patients received Diclofenac suppository one hour prior to the procedure while group “B” did not receive diclofenac suppository. Both groups received 10 ml of intra-urethral plain gel for lubrication during flexible cystoscopy. Pain score was recorded immediately after the procedure using visual analogue scale (VAS). Pre and post procedure pulse rate and systolic blood pressure was also recorded. Statistical analyses were performed using chi-Square test and student t-test. Regression analysis was performed to address the confounding variables.

**RESULTS**: Both groups were comparable for variables including age, duration of procedure, level of operating surgeon and indication of procedure. Most common indication for flexible cystoscopy was removal of double J stent. There was statistically significant difference in the mean pain score between two groups (*p*= 0.012). The difference in post procedure mean pulse rate in the two groups was statistically significant (p= 0.01) however there was no difference observed in mean post procedure systolic blood pressure. Regression analysis showed that none of the confounding variable was significantly affecting the pain perception.

**CONCLUSIONS:** Intra rectal diclofenac suppository is simple and effective pre-emptive analgesia. We recommend its routine use during flexible cystoscopy for better pain control.

Key words: Analgesia; Diclofenac Suppository; diagnosis; flexicystoscopy; office urology

2a) Background:

The earliest reported use of flexible endoscope for examination of bladder neck was by Tsuchida and Sugawara^[1]^. It is now one of the most commonly performed diagnostic as well as therapeutic urologic intervention ^[2]^. Pain associated with cystoscopy varies from patient to patient and there is continuous effort using various methods to reduce pain during and after the procedure to improve patient compliance for flexible cystoscopy. Majority requires local anesthesia or lubricant solution only but some patients may require intravenous sedation^[3]^ or inhalation analgesia (nitrous oxide)^[4]^. Factors contributing to severity of pain include; lubrication, use of topical anesthesia and duration of cystoscopy^[5],^^[6],^^[7]^ but the available evidence for best practice in terms of treatment is continuously evolving^[8]^. The important issues regarding the correct use of intra-urethral gels are, for the most part, left to individual preference^[9]^. Effect of different intra-urethral gels, their dosage, temperature and time of instillation on pain perception has been evaluated in literature. In a randomized control trail, 2% lidocaine gel in two different doses (10 and 20 ml) and plain lubricating gels were found to be equally effective for pain control during flexible cystoscopy *(p=*0.406)^[10]^. Pain perception with use of lidocaine versus plain lubricating gel is less as reported in a meta-analysis by Aaronson et al^[11]^ while another meta-analysis by Patel et al has reported no statistical difference among the two gels for pain control^[12]^. In a study by Komiya et al, oral zaltoprofen has been used as pre-emptive analgesia for rigid cystoscopy and it has been proved to provide better pain control than 2% lidocaine gel alone (11.35 versus 13.69 with a difference of pain score -2.8, p-value 0.0087)^[13]^. Intra-rectal diclofenac suppository administration used by Irer et al has a proven role to reduce pain and improve patients’ tolerance of trans rectal ultrasound-guided prostate biopsy^[14]^.

Diclofenac acts locally and systemically as an anti-inflammatory drug, and it reduces the effects of local mediators involved in the pain response. The diclofenac suppository has a more rapid onset of effect, but a slower rate of absorption than oral enteric-coated tablets. The maximal plasma level occurs within 1 to 2 hours, and this plasma concentration elevation can be maintained for up to 12 hours and that forms the basis of using suppository rather than oral NSAID in our study^[15]^. In the current study we have attempted to assess the use of diclofenac suppository as a preemptive analgesia during flexible ureteroscopy.

2b) Objective:

To compare the difference in pain score during flexible cystoscopy between patients undergoing the procedure with plain gel only and plain gel with diclofenac suppository.

3a) Trial Design:

Single centre, randomized controlled trial with 1:1 randomization. The operating surgeon and assessor of pain score was blinded to the randomization.

3b) Changes to trial design:

Not applicable

4a) Participant:

**Inclusion criteria**:

All male patients above the age of 14 years visiting for

- Evaluation of Haematuria or
- Evaluation of Lower urinary tract **s**ymptoms (which include poor steam of urine, intermittency, hesitancy, incomplete voiding of urine, increase urgency, increase frequency, nocturia and urge incontinence)
- Removal of double J ureteral stent.

**Exclusion criteria**:

- Patients with clinical evidence of urethral stricture and/or prostatitis,
- Patients in which bladder biopsy is planned,
- Patient with psychiatric illness,
- Patient with Asthma as co- morbid
- Patient allergic to NSAIDs (non-steroidal anti-inflammatory drugs)
- Patients who refuse to participate,
- Patients who had flexible cystoscopy before
- Patients with history of chronic analgesia use or
- Patients with language barrier

were excluded.

4b) Study Setting:

This trial was conducted at surgical day care unit of The Aga Khan University Hospital from Feb 2013 to July 2013.

5)Interventions:

Prior to the procedure, patients were explained about Visual Analog Scale (Score zero means no pain and 10 means worst pain). Eligible patients were randomized by a computer-generated list and sealed envelopes. Total of 60 computer-generated sequences were equally divided into two groups. Patients were grouped in Group A (those patients who received diclofenac suppository prior to procedure) and Group B (those patients who did not receive diclofenac suppository prior to procedure). Diclofenac suppository 100 mg was administered per rectally 1 hour prior to the procedure in the pre op area. Both groups received ten ml. of plain lubricating gel immediately before the procedure for the purpose of lubrication.

6a)Outcome:

Procedure was performed by consultant urologist or senior urology resident (residency year 5 and 6) that was blinded to the randomization group. A second resident immediately following the procedure, collected data (pain score) in the operating room. This investigator was blinded to the randomization group (Independent Assessor). Operative time was recorded form the operating room time log. Pre and post procedure pulse rate and blood pressure were recorded for all participants.

6b)Changes to the outcome:

Not applicable

7a)Sample size:

The sample size calculation done by using WHO software taking power=80%, Pain Score in lidocaine only group (13.69) and in Zaltoprofen group (11.35), SD= 2.8 ^(xiii)^

By taking mean difference=13.69- 11.35= 2.34

n= 60

n_1_= 30 in group 1

n_2_= 30 in group 2

7b)Interim analysis and Stopping guidelines:

Since the study was performed over a short period of time, no interim analysis was performed however the study protocol set out a guideline to report the Institutional review board (IRB) and Clinical trial unit (CTU) in case of any adverse event. IRB and CTU were responsible to ensure the conduct of study according to Good Clinical Practice Guidelines.

8a)Randomization: Sequence generation:

Independent pharmacists from CTU dispensed either plain lubricating gel with diclofenac suppository or plain lubricating gel alone, according to a computer generated randomization list.

For allocation of the participants, a computer-generated list of random numbers was used.

8b) Randomization: Type:

Randomization was performed using SPSS 17.0. statistical software with 1:1 random allocation to 1 of 2 treatment groups.

9) Randomization: allocation concealment mechanism :

The plain lubricating gel and diclofenac suppository were dispensed in opaque envelope but since we did not have placebo, the participants were not blinded. However the operating surgeon was blinded to the allocation since both groups received Plain lubricating gel. The assessor of the pain score was also blinded. The allocation concealment was revealed to the primary investigator at the end of study for purpose of analysis.

10) Randomization: implementation: Who generated the allocation sequence, who enrolled participants, and who assigned participants to interventions

Allocation sequence was generated through computer software by the pharmacist from Clinical trial Unit. The patients were assessed by the primary investigator for suitability of requirement in the trial followed by enrollment if eligible. The assignment of the participant to one of two groups was based on the randomization sequence generated through software.

11a)Blinding:

The operating surgeon and the outcome assessor (the one recording pain score and vital signs) were blinded to the intervention.

11b) Similarity of interventions: If relevant, description of the similarity of interventions

We did not use placebo in this study as all intra rectal medications had one or the other effect that can create bias in the assessment of pain. Both groups received plain lubricating gel that was administered by the operating surgeon so the operating surgeon was blinded to the intervention (was not aware that which patient had intra Rectal diclofenac suppository as intervention). The outcome (pain score and vital signs) assessor recorded the readings after the procedure and was blinded to the intervention as well.

12a) Statistical Methods:

Data was analyzed using SPSS^TM^ version 17.0. Results were described in terms of mean and standard deviation for age, duration of procedure and pain score while frequency and percentage were mentioned for categorical variables. The student t-test (independent samples, one-tailed) was used to determine statistical significance of VAS for pain between group A and B.

12b: Additional analysis:

Confounder and effect modifiers i.e. age, level of the person performing procedure, indication for procedure and duration of procedure were analyzed using linear regression analysis. *p*- value of <0.05 was considered as statistically significant.

13a. Participant Flow : For each group, the numbers of participants who were randomly assigned, received intended treatment, and were analyzed for the primary outcome

Seventy-three patients were evaluated for inclusion in the study. Total sixty patients were recruited in the trial and analyzed.

Flow Diagram:

# 13b)Losses and Exclusions: For each group, losses and exclusions after randomisation, together with reasons

None

14a) Recruitment

Age eligible patients were recruited from Feb 2013 to July 2013. Follow up for pain score was done on the day of the procedure.

14b) Reason for stopped trial

Not applicable

15) Baseline data

| Parameters | Group A | Group B | p value |
| --- | --- | --- | --- |
| **Age (years)**  Mean ±SD | 48.53 ± 17.8 | 44.97 ± 14.3 | 0.53 |
| **Duration (min)**  Mean ±SD | 5.76 ± 2,25 | 5.28 ± 2.0 | 0.82 |
| **Indications** |  |  |  |
| JJ stent removal | 17 | 21 |  |
| Evaluation of hematuria | 10 | 6 | 0.497 |
| Evaluation of LUTS | 3 | 3 |  |
| **Level of operating surgeon** |  |  |  |
| Consultant urologist | 2 | 2 |  |
| Senior Urology resident | 28 | 28 | 0.694 |
| **Post procedural Pulse/min**  Mean ±SD | 73.5 ± 4.1 | 76.4 ± 3.8 | **0.01** |
| **Post procedural systolic pressure**  Mean ±SD | 129.3 | 130.1 | 0.15 |
| **Pain score on VAS**  Mean ±SD | 3.16 ± 1.53 | 4.10 ± 1,24 | **0.012** |

16) Number analyzed:

Analysis was performed for all patients who were randomly assigned in each group (30 participants in each group).

17a)Outcomes and estimation

17b) Binary Outcomes:

Not Applicable

18)Ancillary Analysis:

Not performed

19) Harms:

None

20) Limitations:

Small number of participants and lack of placebo were main limitations of the study. Due to strict inclusion criteria it was not possible to recruit large number of participants however with regards to statistics, the study was appropriately powered to examine the research objective. We did not use any placebo as all the intra rectal medications have some effect that can affect the assessment of outcome.

21) Generalizability:

The trial involved adult males (both young and old) undergoing flexible cystoscopy for various indications. Also the procedure was performed by two different levels of operating surgeons. The results indicate that the use of Diclofenac Suppository as a pre-emptive analgesia can help reducing the pain in wide range of patients.

22) Interpretation:

Use of NSAID as pre-emptive has been tried for various surgical procedure^[16],^ ^[17]^. Komiya and co-workers examined the effect of anti-inflammatory drug (NSAID) zaltoprofen that inhibits the generation of prostaglandins as well as the pain induced by bradykinin during rigid cystoscopy^xiii^. The mean age of the patients in their study was 69.3+/- 8.2 (Range: 41-83) while in our study we had relatively younger study subjects (Mean age+/- SD, Range: 46.75+/-16.1 years, 18-80 years) who are presumably more anxious with lower pain threshold. Despite this fact, diclofenac suppository significantly improved the pain perception and proved to be effective regardless of age on regression analysis. Another matter of debate is the statistical method used in the study by Komiya et al^xiii^ where they used “One sample Wilcoxon test” for comparing the two groups which is rather an inappropriate test to demonstrate the effect. In present study we have used regression analysis, which is more stringent method to demonstrate the effect.

23) Registration:

Institutional review board and Clinical trial unit approved the study protocol. It was registered at www.clinicaltrials.gov (ClinicalTrials.gov Identifier: NCT01812928).

24)Protocol:

Full details of the trial protocol can be found in the Supplementary Appendix, available with the full text of this article.

25) Grant:

Grant was received by University Research Committee (URC) Grant Reference number **122010SUR** . The funders had no role in study design, data collection and analysis, decision to publish, or preparation of the manuscript.

**References:**

1. Tsuchida S, Sugawara H. A new flexible fibercystoscope for visualization of the bladder neck. J Urol. 1973 May;109(5):830-1.
2. Beaghler M, Grasso M, 3rd, Loisides P. Inability to pass a urethral catheter: the bedside role of the flexible cystoscope. Urology. 1994 Aug;44(2):268-70.
3. Song YS, Song ES, Kim KJ, Park YH, Ku JH. Midazolam anesthesia during rigid and flexible cystoscopy. Urol Res. 2007 Jun;35(3):139-42.
4. Calleary JG, Masood J, Van-Mallaerts R, Barua JM. Nitrous oxide inhalation to improve patient acceptance and reduce procedure related pain of flexible cystoscopy for men younger than 55 years. J Urol. 2007 Jul;178(1):184-8; discussion 8.
5. Kobayashi T, Nishizawa K, Ogura K. Is instillation of anesthetic gel necessary in flexible cystoscopic examination? A prospective randomized study. Urology. 2003 Jan;61(1):65-8.
6. Kobayashi T, Nishizawa K, Mitsumori K, Ogura K. Instillation of anesthetic gel is no longer necessary in the era of flexible cystoscopy: a crossover study. J Endourol. 2004 Jun;18(5):483-6.
7. Herr HW, Schneider M. Immediate versus delayed outpatient flexible cystoscopy: final report of a randomized study. Can J Urol. 2001 Dec;8(6):1406-8.
8. Soomro KQ, Nasir AR, Ather MH. Impact of patient's self-viewing of flexible cystoscopy on pain using a visual analog scale in a randomized controlled trial. Urology. 2011 Jan;77(1):21-3.
9. Tzortzis V, Gravas S, Melekos MM, de la Rosette JJ. Intraurethral lubricants: a critical literature review and recommendations. J Endourol. 2009 May;23(5):821-6.
10. McFarlane N, Denstedt J, Ganapathy S, Razvi H. Randomized trial of 10 mL and 20 mL of 2% intraurethral lidocaine gel and placebo in men undergoing flexible cystoscopy. J Endourol. 2001 Jun;15(5):541-4.
11. Aaronson DS, Walsh TJ, Smith JF, Davies BJ, Hsieh MH, Konety BR. Meta-analysis: does lidocaine gel before flexible cystoscopy provide pain relief? BJU Int. 2009 Aug;104(4):506-9; discussion 9-10.
12. Patel AR, Jones JS, Babineau D. Lidocaine 2% gel versus plain lubricating gel for pain reduction during flexible cystoscopy: a meta-analysis of prospective, randomized, controlled trials. J Urol. 2008 Mar;179(3):986-90.
13. Komiya A, Endo T, Kobayashi M, Kim W, Araki K, Naya Y, et al. Oral analgesia by non-steroidal anti-inflammatory drug zaltoprofen to manage cystoscopy-related pain: a prospective study. Int J Urol. 2009 Nov;16(11):874-80.
14. Irer B, Gulcu A, Aslan G, Goktay Y, Celebi I. Diclofenac suppository administration in conjunction with lidocaine gel during transrectal ultrasound-guided prostate biopsy: prospective, randomized, placebo-controlled study. Urology. 2005 Oct;66(4):799-802.
15. Idkaidek NM, Amidon GL, Smith DE, Najib NM, Hassan MM. Determination of the population pharmacokinetic parameters of sustained-release and enteric-coated oral formulations, and the suppository formulation of diclofenac sodium by simultaneous data fitting using NONMEM. Biopharm Drug Dispos. 1998 Apr;19(3):169-74.
16. Nagatsuka C, Ichinohe T, Kaneko Y. Preemptive effects of a combination of preoperative diclofenac, butorphanol, and lidocaine on postoperative pain management following orthognathic surgery. Anesth Prog. 2000 Winter;47(4):119-24.
17. Buvanendran A, Kroin JS. Multimodal analgesia for controlling acute postoperative pain. Curr Opin Anaesthesiol. 2009 Oct;22(5):588-93.
