# Supplementary material for: Effect of diclofenac suppository on pain control during flexible cystoscopy-A randomized controlled trial
Source: F1000Res. 2016 Dec 8;5:2834. [Version 1] doi: 10.12688/f1000research.9519.1 (PMC5321120; doi:10.12688/f1000research.9519.1)
Supplement: Supplementary file 2 [file f1000research-5-10254-s0001.tgz › cbb9c8fd-4ae6-400f-8a20-f642167cca6f.zip!/Original Protocol.docx]

Original Research Protocol

Randomized control trail to assess the effect of diclofenac suppository on the pain score during flexible cystoscopy.

**SYNOPSIS**

**TO ASSESS THE EFFICACY OF DICLOFENAC SUPPOSITORY DURING FLEXIBLE CYSTOSCOPY ON PAIN CONTROL in adult males-a double blinded**

**randomized control trial (DUF)**

**Principal Investigator:**

**DR. MEHWASH NADEEM (RESIDENT UROLOGY)**

**SUPERVISOR**

**DR. HAMMAD ATHER (ASSOCIATE PROFESSOR)**

**SECTION OF UROLOGY DEPARTMENT OF SURGERY**

AGA KHAN UNIVERSITY, KARACHI PAKISTAN

**Title**

To assess the efficacy of diclofenac suppository during flexible cystoscopy on pain control.

**Introduction**

Rigid cystoscopy under anesthesia is the gold standard for evaluation of urethra and urinary bladder(1). It may be diagnostic as well as therapeutic, commonly being done for evaluation of haematuria(2), for performing biopsy or resection of urinary bladder mass(3). Other indications are prior to transurethral resection of prostate, removal of foreign bodies and evaluation of urethral stricture (4-5). The rigid cystoscope caused discomfort, especially in males, resulting in suboptimal examination or urethral trauma if performed under sedoanalgesia(6). Lithotomy position is required to allow introduction and adequate manipulation of the cystoscope, which may be uncomfortable and difficult for patients. The rigid instrument may have been inadequate because of lack of urethral compliance (as a result of prior surgery, radiation, or trauma). Fiberoptic technology developed rapidly in the 1960s and 1970s, has led to the development of flexible cystoscopes. The earliest reported use of flexible endoscope for examination of bladder neck was by Tsuchida and Sugawara(7). Since then, Flexible cystoscopy has revolutionized the field of diagnostic urology. Flexible cytoscopy has several advantages over rigid cystoscopy. It can be done under local anaesthesia in the outpatient setting and is thus a highly useful tool, provides an excellent diagnostic view of the bladder; patients have minimal discomfort; and the entire bladder can be visualized in a few minutes. It has diagnostic as well as therapeutic role(8). Pain associated with cystoscopy varies from patient to patient, majority requires local anesthesia or lubricant solution only but some patients may require intravenous sedation(9) or inhalation analgesia (nitrous oxide)(10). During flexible cystoscopy, lubrication, use of topical anesthesia and duration of cystoscopy are recognized as important factors contributing in severity of pain (11-13)but the available evidence for best practice in terms of treatment is continuously

evolving,(14) the important issues regarding the correct use of intraurethral gels are, for the most part, left to individual preference(15). Pain perception with use of lidocaine versus plain lubricating gel is less as reported by Aaronson et al(16) while another meta-analysis by Patel et al has reported no statistical difference among the two gels for pain control(17). In a study by Komiya et al, oral zaltoprofen has been used as pre emptive analgesia for rigid cystoscopy and it has been proved to provide better pain control than 2% lidocaine gel alone (11.35 versus 13.69 with a difference of pain score -2.8, p-value 0.0087) (18). Intrarectal diclofenac suppository administration used by Irer et al has a proven role to reduce pain and improve patients’ tolerance of transrectal ultrasound-guided prostate biopsy(19). Hence use of NSAIDs is not new and it can also be used as an alternate method of pain control during flexible cystoscopy. Diclofenac acts locally and systemically as an anti-inflammatory drug, and it reduces the effects of local

mediators involved in the pain response. The diclofenac suppository has a more rapid onset of effect, but a slower rate of absorption than oral enteric-coated tablets. The maximal plasma level occurs within 1 to 2 hours, and this plasma concentration elevation can be maintained for up to 12 hours and that is the reason for using suppository rather than oral NSAID in our study (20). In

this study we intend to assess and compare the severity of pain during flexible cystoscopy in patients who receive diclofenac suppository along with plain lubricating gel to those who will receive plain lubricating gel only, to see if there is difference in pain score from control group. Thus further reduction of the pain during flexible cystoscopy will be achieved, this will decrease the patients discomfort, will require fewer or no analgesic medication, short duration day care stay, and early return to work. There is no study done previously using diclofenac suppository for pain control during flexible cystoscopy.

**Objective**

To compare mean VAS for pain during flexible cystoscopy between patients undergoing the procedure with plain gel only and with diclofenac suppository besides plain gel.

**Operational Definitions**

**Pain score** will be calculated on Visual Analog Scale which is a 11 digit scale ranging from 0 to

10, Score zero means no pain and 10 means worst pain. Pain score will be recorded immediately after the procedure by resident in operating room.

**Hypothesis**

**Alternative hypothesis** Mean VAS for post-operative pain in patients undergoing flexible cystoscopy with plain lubricating gel only is greater than those undergoing flexible cystoscopy with diclofenac suppository besides plain lubricating gel.

**Material and Methods:**

**Study Design**: Randomized Controlled Trial.

**Setting**: Day care operation suite of the Aga Khan University Hospital. Karachi.

**Duration of Study**: Six months from the date of approval of the synopsis

**Sample Size**: The sample size calculation done by using WHO software taking power=80%, Pain score in K-Y gel only group (13.69) and in diclofenac group (11.35), SD = 2.8, difference. Sample size of 30 patients in each group is required.

**Sampling Technique:** Purposive non-probability sampling

**Sample Selection**

**Inclusion criteria**:

•	**All male patients aged 18 and above**

- Visiting for evaluation of Haematuria or

- Lower urinary tract **s**ymptoms (which include poor steam of urine, intermittency, hesitancy, incomplete voiding of urine, increase urgency, increase frequency, nocturia and urge incontinence)

- For removal of double J ureteral stent will be included in the study.

**Exclusion criteria**:

•	Patients with clinical evidence of urethral stricture and/or prostatitis,

•	Patients in which biopsy will be taken,

•	those having psychiatric illness,

•	Asthmatics

•	Kidney, liver disease

•	Those allergic to NSAIDs (non-steroidal anti-inflammatory drugs)

•	Those who refuse to participate,

•	Having history of chronic analgesia use or

•	Having language barrier will be excluded.

**Data Collection:** All patients, who fulfill the inclusion criteria, will be included in the study. The written consent will be taken by the urology trainee (principal investigator of this study) from the patients after explaining the procedure and study in detail first time in the clinic and then in SDC so that patient will have ample time to think over and make decision whether or not they want to be part of our study. As per GCP guidelines, a copy of the a signed informed consent will be

provided to the patient or the legally acceptable representative. Prior to the procedure, patient will be explained about Visual Analog Scale (Score zero means no pain and 10 means worst pain). Randomization will be performed by clinical trial Unit (CTU) by SPSS statistical softare. After randomization patient will be grouped in Group A (those patients who will receive diclofenac suppository prior to procedure) and Group B (those patients who will not receive diclofenac suppository prior to procedure). Diclofenac suppository 100mg will be administered per rectally 1 hour prior to the procedure by the nurse who will not be blinded. Both groups will receive plain lubricating gel immediately before the procedure for the purpose of lubrication.

Procedure will be performed at surgical day care unit in supine position by consultant urologist or senior urology resident (residency year 5 and 6) that will be blinded to the randomization group. A second resident immediately following the procedure will collect data (pain score) in the operating room. This investigator will be blinded to the randomization group (Independent Assessor). Operative time will be recorded from the operating room time log. Pre and post procedure pulse rate and blood pressure will be recorded for all participants.

The study protocol will be approved by hospital ethical review committee.

**Adverse events/serious adverse events management and reporting:**

The most common side effect is minor allergic reaction which is manifested as swelling of the face, lips, tongue or other parts of the body rash, itching or hives on the skin. Although very few patients may experience shortness of breath or wheezing or difficulty breathing. The patients who are known allergic to any NSAID will be excluded according to the criteria mentioned in

protocol. . The adverse event (AE) will be addressed by the PI immediately and managed with the required medications (IV anti allergic for example Inj. Clemastine and/or Steroids) cost of which will be paid through the grant money. AE, if any noted, will be communicated to the ethical review committee and clinical trial unit within 24 hours of the event according to the GCP guidelines.

**Operative technique and details:**

After scrubbing with povidone-iodine solution and standard draping, plain lubricating gel in volume of 20 ml will be instilled in the urethra. Following which, the urologist or senior urology resident will do the Flexible Cystoscopy using 15 Fr Flexible Digital Cystoscope (Olympus ®). Immediately after the procedure, in operating room, data (pain score) will be collected by

resident. We will quantify pain on Visual Analog Scale (VAS) ranging from 0 score to 10.

DUF trial	Page 7

Patients in which this mentioned analgesic regimen will be found ineffective i.e., VAS of more than 7, will be provided with additional analgesia (Inj. Ketorolac) till the adequate pain control is achieved i.e. VAS of less than 5. Patient with additional requirement of analgesia in either group will be analyzed as sub- group and will not be excluded. Data will be collected on computerized pre made Performa by PI/resident who would be blinded with the treatment group.

**Data Analysis:** Data will be analyzed using SPSS version 16.0. Results will be described in terms of mean and standard deviation for age, duration of procedure and pain score. The student t-test (independent samples, one-tailed) will be used to determine statistical significance of VAS for pain between group A and B. Confounder and effect modifiers i.e. age, level of the person performing procedure and duration of procedure will be analyzed by stratification.

P value <0.05 will be considered significant. Data Storage:

The data collection forms, patients informed consent will be stored in a lock and key with PI to maintain data confidentiality. The data will be stored for 5 years after the completion of the study. The data collection proforma is attached with the synopsis as annexure 1.

**8) Data collection instrument**

Study Subject ID:	Age:

Consultant:	.

Date of Procedure:	.

**Pro forma**

Performed by:	Consultant urologist/	Senior resident

Duration of Procedure:	. Pain score:

Pre Procedure Pulse: Post Procedure Pulse:

Pre Procedure Blood pressure:

Post Procedure Blood Pressure:

**References**

1.	Shah J. Endoscopy through the ages. BJU Int. 2002 May;89(7):645-52.

2.	Keeler B, Ghani KR, Nargund V. Haematuria 1: risk factors, clinical evaluation and laboratory investigations. Br J Hosp Med (Lond). 2007 Aug;68(8):418-22.

3.	Roohullah, Nusrat J, Hamdani SR, Burdy GM, Khurshid A. Cancer urinary bladder--5 year experience at Cenar, Quetta. J Ayub Med Coll Abbottabad. 2001 Apr-

Jun;13(2):14-6.

4.	Akhtar MM, Orakzai N, Qureshi AM. Trans urethral electric vaporisation of

prostate as an alternate to trans urethral resection in benign prostatic hyperplasia. J Ayub

Med Coll Abbottabad. 2004 Oct-Dec;16(4):16-20.

5.	Nabi G, Hemal AK, Khaitan A. Endoscopic management of an unusual foreign body in the urinary bladder leading to intractable symptoms. Int Urol Nephrol.

2001;33(2):351-2.

6.	Goldfischer	ER, Cromie	WJ, Karrison TG, Naszkiewicz	L, Gerber	GS.

Randomized, prospective, double-blind study of the effects on pain perception of lidocaine jelly versus plain lubricant during outpatient rigid cystoscopy. J Urol. 1997

Jan;157(1):90-4.

7.	Tsuchida S, Sugawara H. A new flexible fibercystoscope for visualization of the

bladder neck. J Urol. 1973 May;109(5):830-1.

8.	Beaghler M, Grasso M, 3rd, Loisides P. Inability to pass a urethral catheter: the

bedside role of the flexible cystoscope. Urology. 1994 Aug;44(2):268-70.

9.	Song YS, Song ES, Kim KJ, Park YH, Ku JH. Midazolam anesthesia during rigid

and flexible cystoscopy. Urol Res. 2007 Jun;35(3):139-42.

10.	Calleary JG, Masood J, Van-Mallaerts R, Barua JM. Nitrous oxide inhalation to

improve patient acceptance and reduce procedure related pain of flexible cystoscopy for men younger than 55 years. J Urol. 2007 Jul;178(1):184-8; discussion 8.

11.	Kobayashi T, Nishizawa K, Ogura K. Is instillation of anesthetic gel necessary in flexible cystoscopic examination? A prospective randomized study. Urology. 2003

Jan;61(1):65-8.

12.	Kobayashi T, Nishizawa K, Mitsumori K, Ogura K. Instillation of anesthetic gel

is no longer necessary in the era of flexible cystoscopy: a crossover study. J Endourol.

2004 Jun;18(5):483-6.

13.	Herr HW, Schneider M. Immediate versus delayed outpatient flexible cystoscopy:

final report of a randomized study. Can J Urol. 2001 Dec;8(6):1406-8.

14.	Soomro KQ, Nasir AR, Ather MH. Impact of patient's self-viewing of flexible cystoscopy on pain using a visual analog scale in a randomized controlled trial. Urology.

2011 Jan;77(1):21-3.

15.	Tzortzis V, Gravas S, Melekos MM, de la Rosette JJ. Intraurethral lubricants: a

critical literature review and recommendations. J Endourol. 2009 May;23(5):821-6.

16.	Aaronson DS, Walsh TJ, Smith JF, Davies BJ, Hsieh MH, Konety BR. Meta-

analysis: does lidocaine gel before flexible cystoscopy provide pain relief? BJU Int. 2009

Aug;104(4):506-9; discussion 9-10.

17. Patel AR, Jones JS, Babineau D. Lidocaine 2% gel versus plain lubricating gel for pain reduction during flexible cystoscopy: a meta-analysis of prospective, randomized, controlled trials. J Urol. 2008 Mar;179(3):986-90.

18. Komiya A, Endo T, Kobayashi M, Kim W, Araki K, Naya Y, et al. Oral analgesia

by non-steroidal anti-inflammatory drug zaltoprofen to manage cystoscopy-related pain:

a prospective study. Int J Urol. 2009 Nov;16(11):874-80.

19. Irer B, Gulcu A, Aslan G, Goktay Y, Celebi I. Diclofenac suppository administration in conjunction with lidocaine gel during transrectal ultrasound-guided prostate biopsy: prospective, randomized, placebo-controlled study. Urology. 2005

Oct;66(4):799-802.

20. Idkaidek NM, Amidon GL, Smith DE, Najib NM, Hassan MM. Determination of the population pharmacokinetic parameters of sustained-release and enteric-coated oral formulations, and the suppository formulation of diclofenac sodium by simultaneous data fitting using NONMEM. Biopharm Drug Dispos. 1998 Apr;19(3):169-74.
