# Supplementary material for: Effect of diclofenac suppository on pain control during flexible cystoscopy-A randomized controlled trial
Source: F1000Res. 2016 Dec 8;5:2834. [Version 1] doi: 10.12688/f1000research.9519.1 (PMC5321120; doi:10.12688/f1000research.9519.1)
Supplement: Peer review history

# Peer review: Referee response for version 1

**DOI:** 10.5256/f1000research.10254.r20345

**Published:** 2017-02-17

**Authors:** Michael Chrisofos

**Affiliations:** 1 2nd Department of Urology, Sismanoglio General Hospital, National and Kapodistrian University of Athens, Athens, Greece

Usage of diclofenac suppository, seems to be an excellent choice, based on the results of this revelatory research, so I congratulate the authors for running a well-designed trial and obtained an acceptable scientific standard.

 The issue of non-anesthetists procedures, for any examination like flexible cystoscopy, is always the most common matter of concern about patients for the practicing Urologists. Worldwide there are many used local anesthetic drugs or substitutes transient analgesia, without much success in the best case or side effects to their use in the worst. However, the use of an analgesic which may be readily obtained from the patient while avoiding adverse effects associated with the gastric tolerability, for maintaining a sufficiently efficient to meet the requirements as a pain reliever during flexible cystoscopy, is not only necessary but also imperative. We will certainly take into account the fact that during both rigid and flexible cystoscopy, there is a potential of greater momentary discomfort when the stent is being pulled through the bladder neck and due to the length of the urethra. So, an effective analgesic, not only will help the patient to be well tolerated, it will give the advantage to the urologist to deal with more calm the cystoscopy.

 Moreover it would be interesting to know the authors’ feedback on the response of patients according to sex and age in the administration of diclofenac.

I have read this submission. I believe that I have an appropriate level of expertise to confirm that it is of an acceptable scientific standard.

# Peer review: Referee response for version 1

**DOI:** 10.5256/f1000research.10254.r18368

**Published:** 2016-12-19

**Authors:** Noor NP Buchholz, Krishanu Das

**Affiliations:** 1 U-merge (Urology in Emerging Countries), London, UK; 2 Royal Adelaide Hospital, Adelaide, SA, Australia

K Das and N Buchholz: Comment on “Nadeem M and Ather MH. Effect of diclofenac suppository on pain control during flexible cystoscopy-A randomized controlled trial [version 1; referees: awaiting peer review]. F1000Research 2016, 5:2834 (doi: 10.12688/f1000research.9519.1)”

 Firstly, I congratulate the authors for a well-chosen study topic, and running a well-designed trial. Flexible cystoscopy, being a commonly practised office procedure without the involvement of anesthetists on most occasions, the choice of appropriate analgesics always remains a dilemma to the practising urologist. A gamut of approaches ranging from local anaethetic usage, lubricant solution to usage of inhalational agents has been practiced with mixed reports. Usage of diclofenac suppository, as has been advocated in this study appears to be a simple and effective approach for conducting this procedure. The statistics supports the claim of the proponents regarding the efficacy of this drug. Considering the ease of administration it will be a good option for the practising urologist conducting office flexible cystoscopy.

 It would be interesting to know the authors’ feedback on the following aspects from their experience in this study-

1. Was there a difference in pain perception in individuals undergoing diagnostic flexible cystoscopy and those undergoing flexible cystoscopy for stent removal as the latter often involves added pain stimulus to the patient due to additional instrumentation.
2. Was the benefit of diclofenac suppository equally perceived in males and females - our personal observations are flexible cystoscopies are presumably easier and well tolerated in females than males.
3. The last sentence in the introduction says- “In the current study we have attempted to assess the use of diclofenac suppository as a pre-emptive analgesia during flexible ureteroscopy” - we presume this is a typographical error and should be “flexible cystoscopy” instead of flexible ureteroscopy – this needs to be corrected if so.

We have read this submission. We believe that we have an appropriate level of expertise to confirm that it is of an acceptable scientific standard.

# Response

**Published:** 2016-12-25

**Authors:** M Hammad Ather

**Affiliations:** Department of Surgery, Aga Khan University, Pakistan

We thank Drs. Buccholz and Das for their valuable comments on the above submission. In response to the questions raised:

1. No we did not find any difference in the diagnostic cystoscopy versus interventional flexicystoscopy(JJ stent removal). The subgroup analysis not shared in the data (as the numbers were small) was insignificant, however, our own experience was that there is indeed is a momentary discomfort when the stent is being pulled through the bladder neck.
2. Again an important point as indeed due to the length of the urethra there is a potential of greater discomfort in men during flexible cystoscopy. We, however, notice no significant difference in our practice (perhaps due to routine use of low pressure irrigation during introduction of the scope to keep the urethra open.) However, in our current work we only studied men.
3. Indeed it is a typographical error, many thanks for pointing this out.
